# Supplementary material for: Immunohistochemistry-Based Taxonomical Classification of Bladder Cancer Predicts Response to Neoadjuvant Chemotherapy
Source: Cancers (Basel). 2020 Jul 3;12(7):1784. doi: 10.3390/cancers12071784 (PMC7408104; doi:10.3390/cancers12071784)
Supplement: Supplementary file 1 [file cancers-12-01784-s001.pdf]

Immunohistochemistry-based Taxonomical Classification of Bladder Cancer Predicts Response to Neoadjuvant Chemotherapy

Supplementary material

Supplementary Tables 1-5

Supplementary Figures 1-5

Supplementary Table 1. Characteristics of the subjects included and excluded from the analyses.

Characteristics	All N = 215	Excluded N = 83	Included* N = 132	P
Centre				0.00016
HUGTiP	168 (78.1%)	76 (91.6%)	92 (69.7%)	
Manresa	47 (21.9%)	7 (8.43%)	40 (30.3%)	
Sex				0.33
M	199 (92.6%)	75 (90.4%)	124 (93.9%)	
F	16 (7.44%)	8 (9.64%)	8 (6.06%)	
Age (cont)	66.0 [59.0;71.5]	65.0 [58.0;70.5]	66.0 [61.0;72.0]	0.34
Age (tertiles, years)				0.25
[35,62]	76 (35.3%)	35 (42.2%)	41 (31.1%)	
[62,69]	72 (33.5%)	25 (30.1%)	47 (35.6%)	
[69,83]	67 (31.2%)	23 (27.7%)	44 (33.3%)	
Morphology				0.37
Urothelial	183 (85.1%)	70 (84.3%)	113 (85.6%)	
Mixed	23 (10.7%)	9 (10.8%)	14 (10.6%)	
Adenocarcinoma	6 (2.79%)	4 (4.82%)	2 (1.52%)	
Other	3 (1.40%)	0 (0.00%)	3 (2.27%)	
Lymphovascular invasion				0.261
No	188 (87.4%)	74 (89.2%)	114 (86.4%)	
Yes	24 (11.2%)	7 (8.43%)	17 (12.9%)	
Unknown	1 (0.47%)	1 (1.20%)	0 (0.00%)	
‘Missing’	2 (0.93%)	1 (1.20%)	1 (0.76%)	
Grade				0.55
Low	6 (2.79%)	3 (3.61%)	3 (2.27%)	
High	208 (96.7%)	79 (95.2%)	129 (97.7%)	
'Missing'	1 (0.47%)	1 (1.20%)	0 (0.00%)	
cTNM				
0-3	16 (7.44%)	3 (3.61%)	13 (9.85%)	0.16
4-6	157 (73.0%)	61 (73.5%)	96 (72.7%)	
7+	41 (19.1%)	19 (22.9%)	22 (16.7%)	
'Missing'	1 (0.47%)	0 (0.00%)	1 (0.76%)	
pTNM:				0.73
Not assessable	9 (4.19%)	8 (9.64%)	1 (0.76%)	
Complete	58 (27.0%)	18 (21.7%)	40 (30.3%)	
Partial	27 (12.6%)	10 (12.0%)	17 (12.9%)	
Not responder	117 (54.4%)	43 (51.8%)	74 (56.1%)	
'Missing'	4 (1.86%)	4 (4.82%)	0 (0.00%)	
Lymph node involvement				0.04
No	161 (74.9%)	59 (71.1%)	102 (77.3%)	
Yes	29 (13.5%)	5 (6.02%)	24 (18.2%)	
'Missing'	25 (11.6%)	19 (22.9%)	6 (4.55%)	
Lymph nodes resected				0.18
0-9	112 (52.1%)	42 (50.6%)	70 (53.0%)	
10+	78 (36.3%)	22 (26.5%)	56 (42.4%)	

Characteristics	All N = 215	Excluded N = 83	Included* N = 132	P
'Missing'	25 (11.6%)	19 (22.9%)	6 (4.55%)	
Treatment				0.04
CMV	66 (30.7%)	34 (41.0%)	32 (24.2%)	
CG	122 (56.7%)	38 (45.8%)	84 (63.6%)	
CaG	20 (9.30%)	8 (9.64%)	12 (9.09%)	
Other**	5 (2.33%)	1 (1.20%)	4 (3.03%)	
'Missing'	2 (0.93%)	2 (2.41%)	0 (0.00%)	

*This group includes 6 cases from whom tumour tissue was used for TMA construction but results for all 4 IHC markers were not available. **Other: Dose-dense MVAC.

Supplementary Table 2. Patient characteristics according to the response to treatment (N = 131)*.

Characteristics	Complete N = 40	Partial/ Non-responders N = 91	*P* value
**Centre**			
Can Ruti	28 (70.0%)	64 (70.3%)	1.000
Manresa	12 (30.0%)	27 (29.7%)	
**Sex**			
M	39 (97.5%)	84 (92.3%)	0.442
F	1 (2.50%)	7 (7.69%)	
**Age** (cont.), median [1st-3rd quartile]	66.0 [62.8;69.8]	67.0 [60.5; 72.0]	0.777
**Age** (tertiles, years)			
[35,62]	10 (25.0%)	31 (34.1%)	0.067
[62,69]	20 (50.0%)	26 (28.6%)	
[69,83]	10 (25.0%)	34 (37.4%)	
**Morphology**			
Urothelial	37 (92.5%)	75 (82.4%)	0.465
Mixed	3 (7.50%)	11 (12.1%)	
Adenocarcinoma	0 (0.00%)	2 (2.20%)	
Other	0 (0.00%)	3 (3.30%)	
**Lymphovascular invasion**			
No	36 (90.0%)	77 (84.6%)	0.568
Yes	4 (10.0%)	13 (14.3%)	
Unknown	0 (0.00%)	1 (1.10%)	
**Grade**			
Low	1 (2.50%)	2 (2.20%)	1.000
High	39 (97.5%)	89 (97.8%)	
**cTNM**			
0–3	6 (15.0%)	6 (6.59%)	0.303
4–6	27 (67.5%)	69 (75.8%)	
7+	6 (15.0%)	16 (17.6%)	
'Missing'	1 (2.50%)	0 (0.00%)	
**Lymph node involvement**			
No	38 (95.0%)	63 (69.2%)	0.002
Yes	0 (0.00%)	24 (26.4%)	
'Missing'	2 (5.00%)	4 (4.40%)	
**Lymph nodes resected**			
0–9	19 (47.5%)	51 (56.0%)	0.449
10+	19 (47.5%)	36 (39.6%)	
'Missing'	2 (5.00%)	4 (4.40%)	
**Hydronephrosis**			
No	27 (67.5%)	52 (57.1%)	0.339
Yes	13 (32.5%)	39 (42.9%)	
**Treatment**			
CG	25 (62.5%)	58 (63.7%)	1.000
CaG	4 (10.0%)	8 (8.79%)	
CMV	10 (25.0%)	22 (24.2%)	
'Missing'	1 (2.50%)	3 (3.30%)	

*For one patient, response could not be assessed.

Supplementary Table 3. Distribution of bladder cancer gene mutations according to the 3 clusters identified (N = 126).

MARKER	MIXED N = 35	LUMINAL-LIKE N = 44	BASAL-LIKE N = 47	*P* VALUE
FGFR3				0.015
wt	23 (69.7%)	35 (79.5%)	44 (93.6%)	
mut	8 (24.2%)	4 (9.09%)	1 (2.13%)	
'Missing'	2 (6.06%)	5 (11.4%)	2 (4.26%)	
PIK3CA				0.132
wt	21 (63.6%)	35 (79.5%)	35 (77.8%)	
mut	7 (21.2%)	3 (6.82%)	2 (4.44%)	
'Missing'	5 (15.2%)	6 (13.6%)	8 (17.8%)	
HRAS				0.314
wt	13 (39.4%)	23 (54.8%)	19 (42.2%)	
mut	1 (3.03%)	2 (4.76%)	0 (0.00%)	
'Missing'	19 (57.6%)	17 (40.5%)	26 (57.8%)	
KRAS				0.131
wt	14 (42.4%)	25 (58.1%)	16 (35.6%)	
mut	0 (0.00%)	1 (2.33%)	3 (6.67%)	
'Missing'	19 (57.6%)	17 (39.5%)	26 (57.8%)	
NRAS				0.241
wt	13 (39.4%)	26 (60.5%)	18 (40.0%)	
mut	1 (3.03%)	0 (0.00%)	1 (2.22%)	
'Missing'	19 (57.6%)	17 (39.5%)	26 (57.8%)	
RAS-any				0.415
wt	12 (36.4%)	22 (52.4%)	15 (33.3%)	
mut	2 (6.06%)	3 (7.14%)	4 (8.89%)	
'Missing'	19 (57.6%)	17 (40.5%)	26 (57.8%)	

Supplementary Table 4. Predictive factors of complete response to neoadjuvant treatment. Multivariate logistic regression models (N = 126). Model 1 and Model 2 differ regarding adjustment for by lymphovascular and lymph node invasion.

Factors	Model 1			Model 2		
	OR	*P* value	95% CI	OR	*P* value	95% CI
Morphology						
Urothelial	Ref			Ref		
Mixed	0.27	0.083	(0.06, 1.19)	0.32	0.159	(0.06, 1.56)
Lymphovascular invasion						
No	-			Ref.		
Yes	-			0.61	0.531	(0.13, 2.90)
Lymph node invasion						
No	-			Ref		
Yes	-			0.07	0.014	(0.00, Inf)
cTNM						
T2 N0	Ref			Ref		
T3/4 N0	0.31	0.121	(0.07, 1.37)	0.22	0.093	(0.04, 1.28)
TxN1Mx	0.36	0.245	(0.06, 2.01)	0.33	0.293	(0.04, 2.59)
Clusters						
Mixed	Ref			Ref		
Luminal-like cluster	1.28	0.699	(0.37, 4.49)	1.34	0.672	(0.34, 5.26)
BASQ-like cluster	3.96	0.017	(1.28, 12.20)	4.06	0.026	(1.18, 13.99)

Both models were adjusted for age and centre.

Supplementary Table 5. Predictive factors of pCR to NAC. Multivariate logistic regression models (N = 100) *.

Factor	Model 1		Model 2	
	**OR**	**P value**	**OR**	**P value**
Age (years)				
<62	Ref		Ref	
62-69	2.10	0.186	2.16	0.206
70-83	0.69	0.560	0.76	0.693
Morphology				
Urothelial	Ref		Ref	
Mixed	0.43	0.310	0.54	0.471
Lymphovascular invasion				
No	–	–	Ref	
Yes	–	–	1.38	0.723
Lymph node involvement				
No	–	–	Ref	
Yes	–	–	0.08	0.03
cTNM				
T2 N0	Ref		Ref	
T3/4 N0	0.33	0.190	0.19	0.093
Clusters				
Mixed	Ref		Ref	
Luminal-like cluster	0.93	0.916	0.81	0.790
BASQ-like cluster	3.22	0.062	3.28	0.076

* Patients treated with Carboplatin or with lymph node involvement were excluded from the analyses. Both models were adjusted for centre.

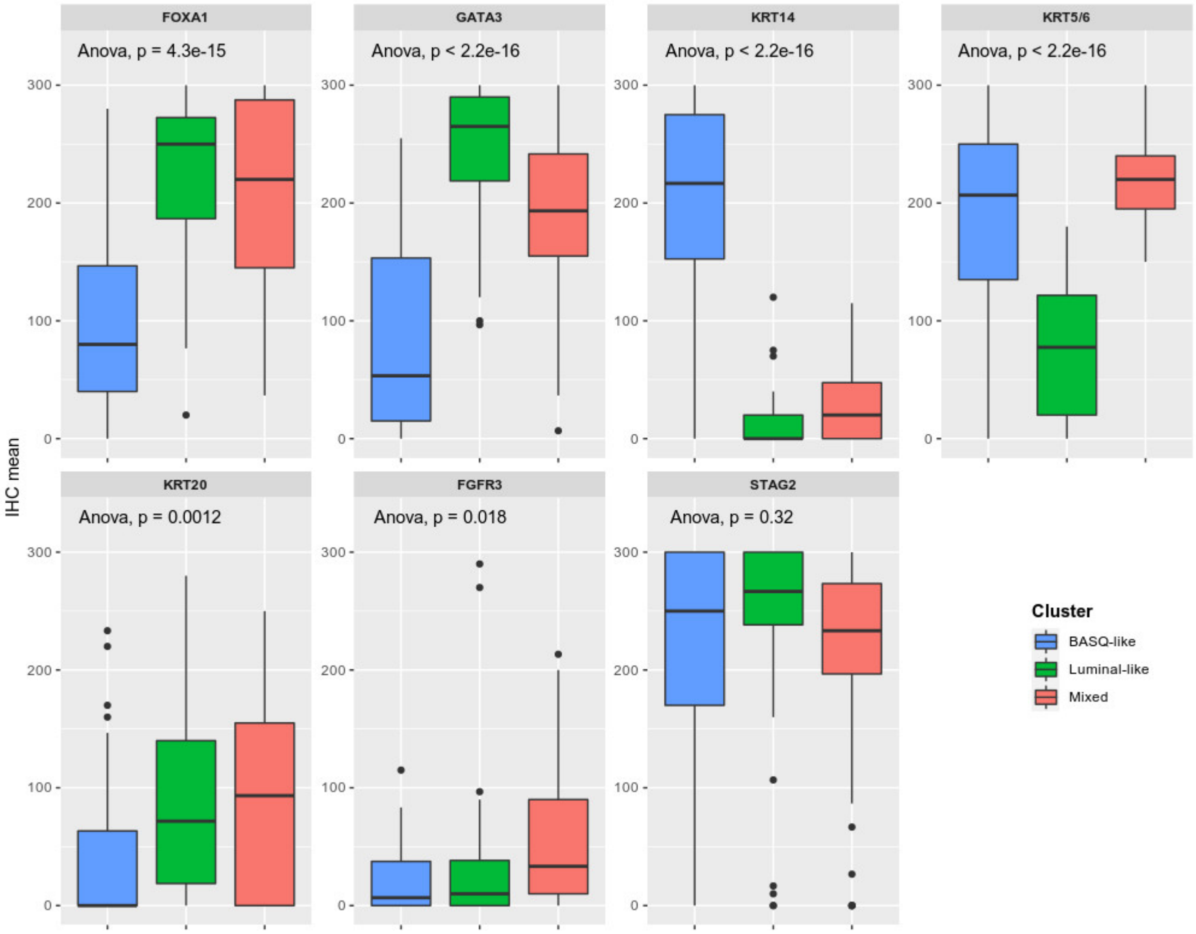

Supplementary Figure 1. Median and interquartile range [IQR] of HS corresponding to each tumour marker according to the 3 clusters identified (N = 126).

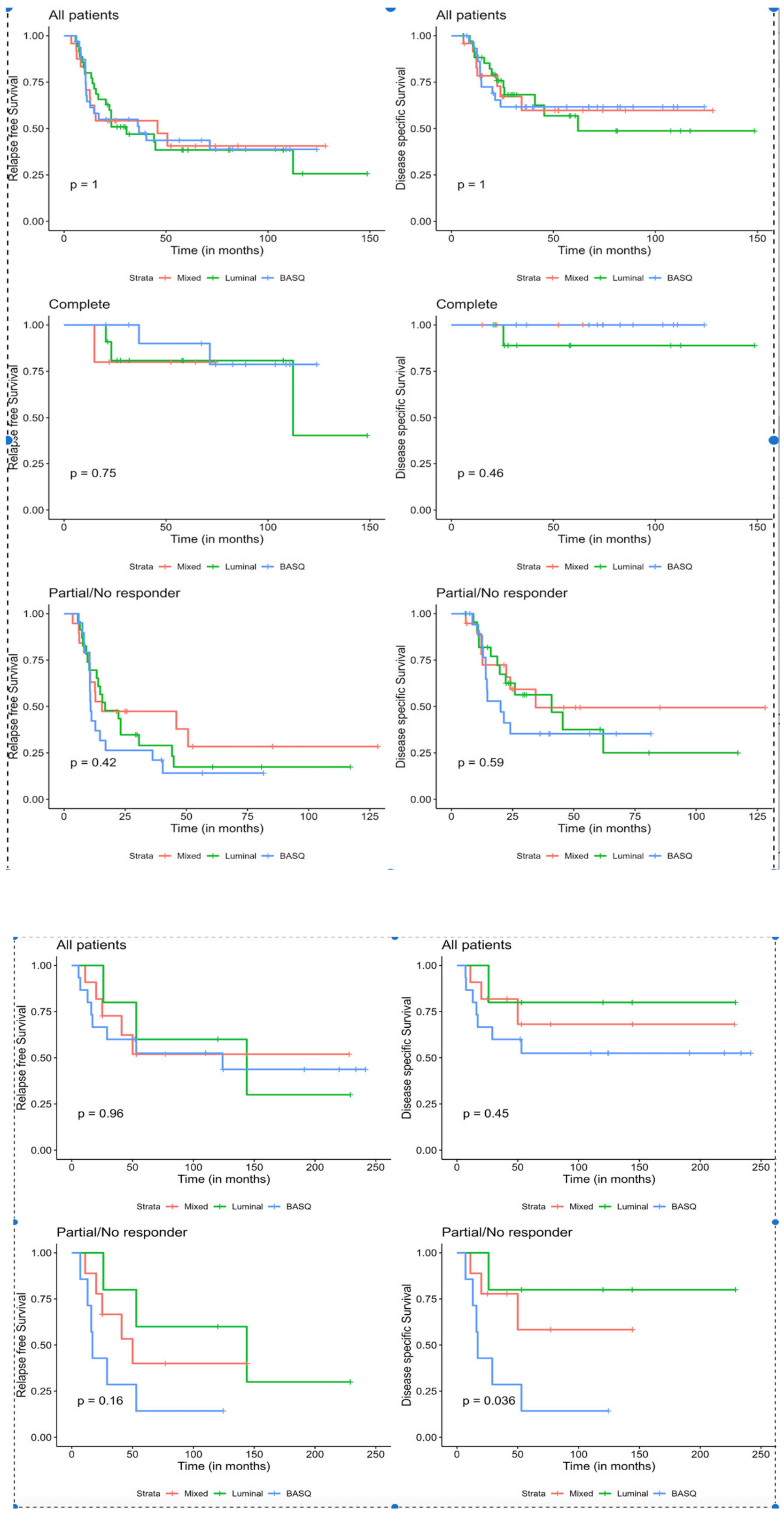

Supplementary Figure 2. Kaplan-Meier relapse-free survival (left panels) and disease-specific survival (right panels) curves for patients treated with CG/CaG (A) vs. CMV (B) combination therapy. While sample size did not allow stratified analyses, we found a similar trend in the association of BASQ subtype with outcome in both patient subgroups..

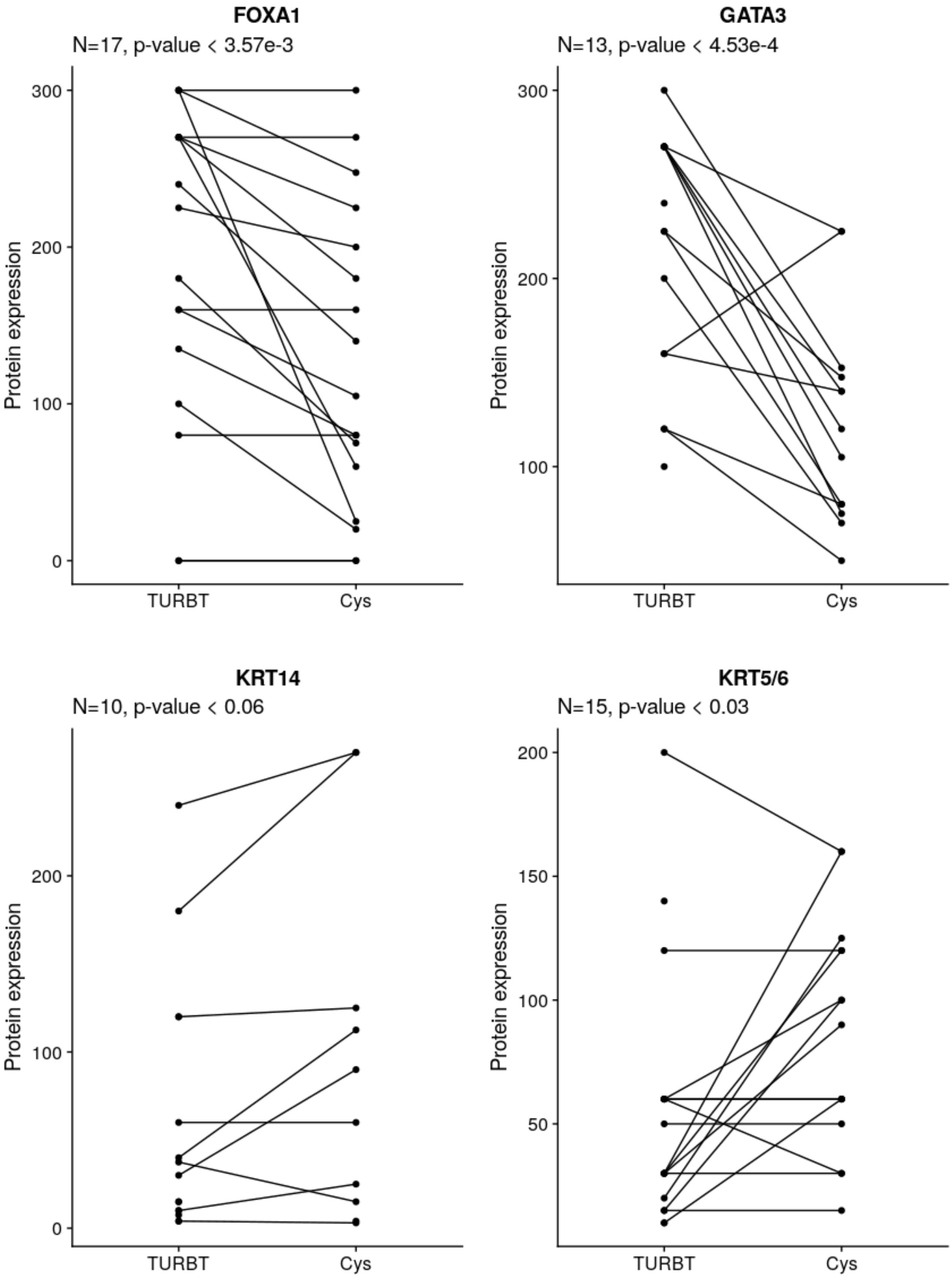

Supplementary Figure 3. Comparison of marker expression in full sections of samples obtained at TURBT and cystectomy (Cys) from patients treated with NAC. Statistical analysis: repeated measurements ANOVA.

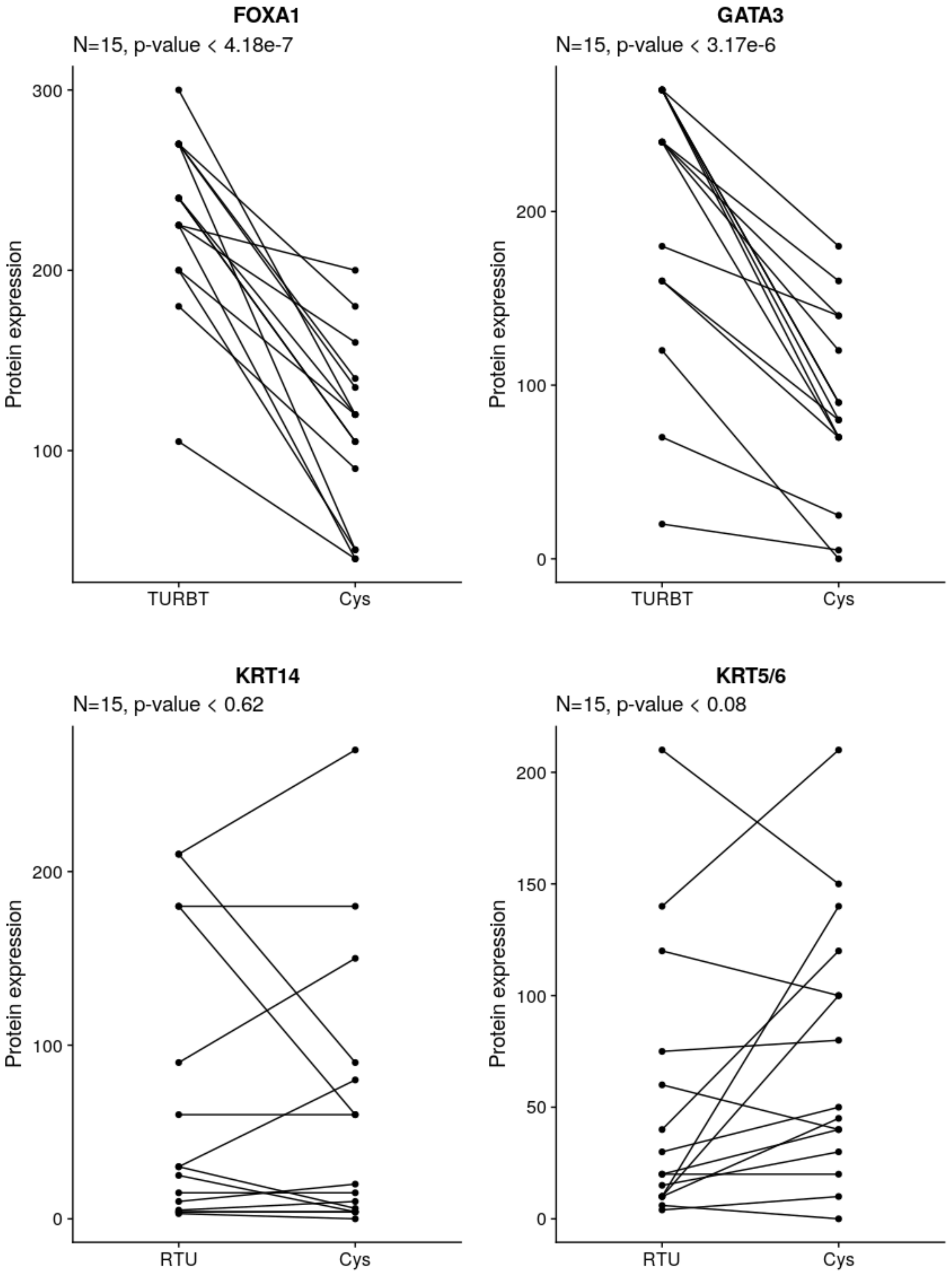

Supplementary Figure 4. Comparison of marker expression in full sections of samples obtained at TURBT and cystectomy (Cys) from patients who did not receive NAC. Statistical analysis: repeated measurements ANOVA.

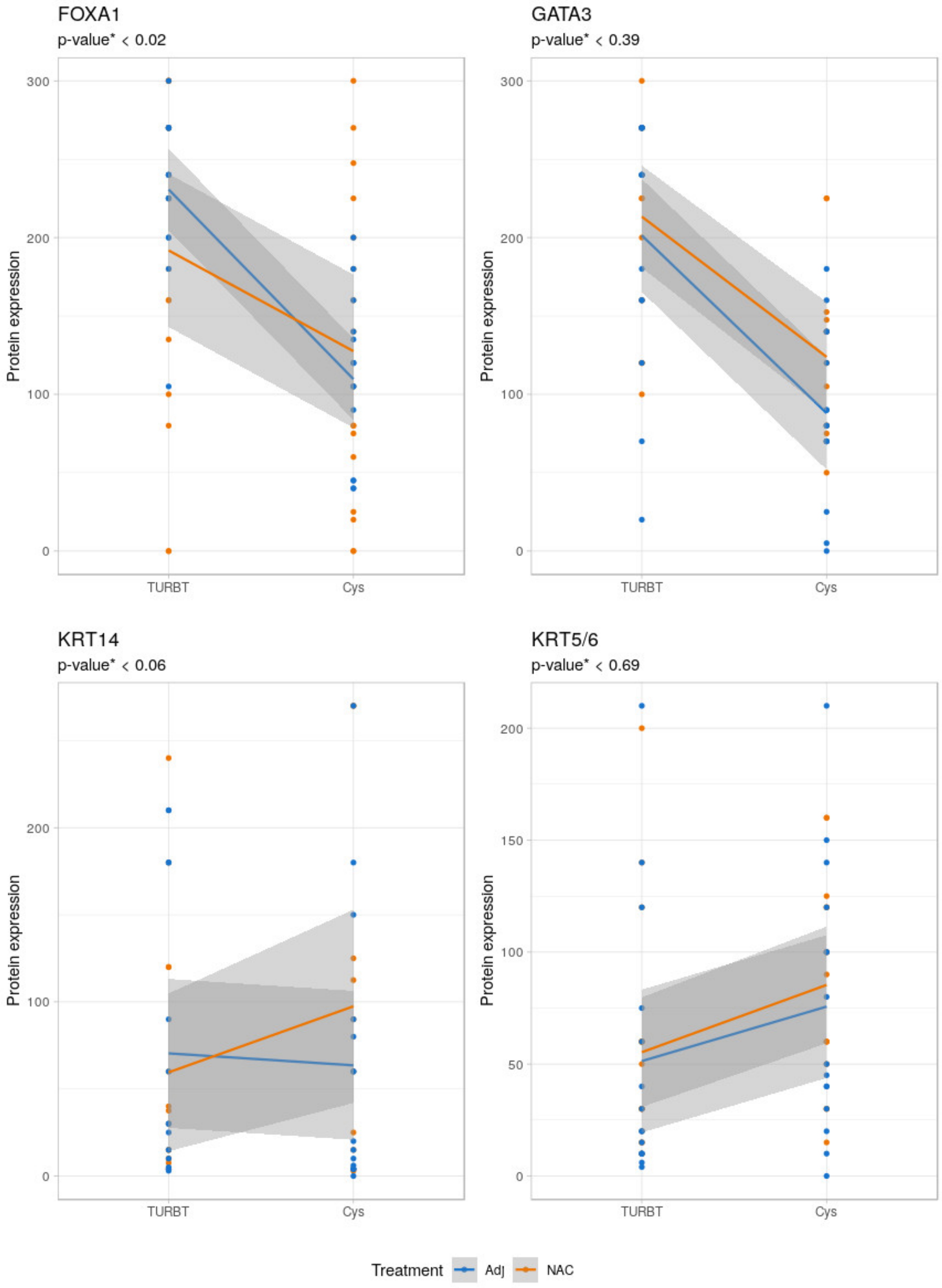

Supplementary Figure 5. Interaction analysis of marker expression in full sections of samples obtained at TURBT and cystectomy (Cys) from patients treated (orange) or not (blue) with NAC. Statistical analysis: linear mixed effects model; shown is the interaction P-value.
